# Explaining bioenergy: representations of jatropha in Kenya before and after disappointing results

**DOI:** 10.1186/s40064-016-3687-y

**Published:** 2016-11-22

**Authors:** Carol Hunsberger

**Affiliations:** Department of Geography, The University of Western Ontario, London, ON N6A5C2 Canada

**Keywords:** Biofuel, *Jatropha curcas*, Discourse, Responsible innovation, Kenya

## Abstract

Proponents of *Jatropha curcas* portrayed the crop as a ‘sustainable biofuel’ that was less threatening to food security and forests than other energy crops, creating a reputation that helped jatropha projects to multiply quickly throughout the global South. However, many jatropha initiatives failed to thrive and ultimately collapsed. This paper investigates how actors involved with jatropha in Kenya explained their visions of bioenergy at two points in time. In 2009, when many activities were beginning, I interviewed small-scale farmers, NGO staff, researchers, donors, government officials and members of the private sector about their expectations of jatropha as an energy crop. In late 2013, after jatropha activities in the country had dwindled, I re-interviewed many of the same individuals about their current views and their explanations of the events that had transpired since the initial fieldwork. Synthesizing these two sets of representations provides insight into how biofuel projects have been constructed, negotiated and renegotiated. Early hopes for jatropha rested on the belief that it could achieve many goals simultaneously, but when it failed to meet expectations proponents chose between two strategies: (1) ‘unbundling’ these goals to pursue separately the various aspirations they had initially attached to jatropha; and (2) seeking a new means of achieving the same bundle of goals. Understanding the choices made by jatropha actors in Kenya contributes to knowledge on the political ecology of biofuels and responsible innovation, and may signal patterns to come as even greater expectations are attached to multi-use feedstocks in pursuit of the bioeconomy.

## Background

What happens after a ‘miracle crop’ falls from grace? A decade ago the oilseed shrub *Jatropha curcas* (hereafter jatropha) rose to fame, buoyed by hopes that it would provide a source of biofuel while growing in conditions unsuitable for food production. Today there is an emerging consensus that jatropha projects throughout the global South failed to meet these high expectations—and in most cases, modest ones as well (see for example Neimark 2016; Ariza-Montobbio and Lele 2010; German et al. 2011a; Romijn et al. 2014). This paper looks closely at the explanations that key actors have offered while navigating the life course of an untested innovation. Focusing on jatropha in Kenya, I compare statements made by key actors—NGO, government, donor and private sector representatives—during and after a period of strong enthusiasm for jatropha projects. By investigating how these informants’ representations of jatropha changed over time, I aim to explore how representations of jatropha were revised and repurposed after disappointing results. Understanding these discursive shifts contributes to conversations about the relationship between discourse and lived experience for new innovations, as well as when and how to attribute responsibility when such innovations go wrong.

Discourse plays a major role in pushing forward new ‘development’ initiatives. Discursive representations^1^ shape desires as well as understandings of problems and solutions; they call possibilities into being. As Jessop (2005) explains in relation to ‘economic imaginaries’, “discourses are performative rather than purely descriptive” (142)—they have material consequences, sometimes creating the very economic conditions they predict. Tsing’s (2000) ‘economy of appearances’ concept similarly shows how ‘myth’ and ‘spectacle’ are used to attract investment for new ventures; the viability of a new enterprise often depends on how successfully its promoters can persuade funders that lucrative benefits will ensue. Fairhead et al. (2012) further explore the materiality of anticipation through the idea of ‘discursive commodities’—objects that are traded in the present even though they do not yet exist; they have market value based on expectations about the future. Together, these contributions highlight how strongly discursive representations—especially of new or unproven phenomena—can shape economic, social and ecological realities, for example by influencing demand, price signals, policies, and donor or investor decisions.

Biofuels have been the subject of active discursive battles with material consequences. For a time, biofuels were attached to a ‘win–win–win’ discourse of mitigating climate change, improving energy security and promoting rural development (Franco et al. 2010). Small-scale biofuel projects and those based on crop residues or mixed cropping systems were particularly praised for their potential to support livelihoods, provide energy and protect the environment (Tilman et al. 2009; Milder et al. 2008). A wave of biofuel policies stimulated production and markets (Bailis and Baka 2011). But this optimistic vision has been aggressively challenged: biofuels have been criticized for their actual and potential impacts on food security, local ecologies, livelihoods, and their climate change mitigation potential (Houtart 2010; Fargione et al. 2010; Melillo et al. 2009; Immerzeel et al. 2014; German et al. 2011b). In response to such concerns, several governments have modified their policies to include new guidelines for ‘sustainable biofuels’ (Hunsberger et al. 2014; German and Schoneveld 2012). Recent pro-biofuel discourses draw on the idea that the most common sources of biofuel offer solutions to many problems because they are ‘flex crops’—crops that can be used to make food, feed, fuel, and commercial and/or industrial products (Borras et al. 2015) while also playing a variety of social and ecological roles. For example, industry representatives have portrayed oil palm as an efficient source of food and clean energy, a driver of economic development, and a tropical tree that stores carbon and improves biodiversity—all in one (Hunsberger and Alonso-Fradejas 2016).

*Jatropha curcas*, a shrub indigenous to Latin America that has spread widely throughout the global South, benefited from having a unique reputation among sources of biofuel. Jatropha was portrayed as less of a threat to food security and forests than other energy crops—and therefore as a ‘sustainable biofuel’—for three reasons. First, since it is inedible, jatropha would not directly divert food supplies to produce fuel; second, by growing in harsh environments, it would not compete with food crops for arable land; and third, because it is not eaten by animals, jatropha could act as a living fence to protect food crops against livestock and wildlife (Achten et al. 2014). These claims helped jatropha to withstand a general backlash against biofuels and perhaps also to deflect specific criticism, particularly from international NGOs (for example Friends of the Earth 2009, 2010; WWF 2009). While jatropha appears to be less flexible than oil palm because it cannot be used as food or livestock feed, its proponents have nonetheless bundled together expectations that jatropha can help mitigate climate change, provide energy, improve soil health, boost farmer incomes, improve food security and stimulate economic activity at various scales (Hunsberger and Alonso-Fradejas 2016).

Much attention has recently focused on the buildup and subsequent deflation of a jatropha ‘hype’—a surge of interest and expectations (e.g. Achten et al. 2014; Romijn et al. 2014; Simandjuntak 2014; Afiff 2014). Despite extensive analysis of how this ‘hype’ remained unrealized, little research so far has examined how key actors themselves have explained jatropha’s trajectory, both before and after disappointing results. Focusing on the case of Kenya, this paper seeks to fill this gap by addressing two questions: (1) How did individuals who were involved with early efforts to promote jatropha explain the crop’s story—past, present and future—when early initiatives failed to live up to expectations? And, (2) How did their representations of jatropha compare over time?

Answering these questions for a particular crop in a particular country can contribute to an improved understanding of how wider biofuel projects are constructed, negotiated and renegotiated—an important task given the uneven social outcomes of biofuel production to date. More broadly, understanding the discursive patterns used to promote and protect untested development interventions can provide tools to critically assess such projects, detect earlier when an innovation may be heading in a harmful direction, and begin to address questions of who should take what forms of responsibility for the impacts of such innovations.

## Methods

This project began as an exploration of the politics of development surrounding jatropha in Kenya. Using a qualitative case study approach, from January–July 2009 I conducted in-depth, semi-structured interviews with key stakeholders—representatives of government, NGOs, the private sector, donors and researchers—as well as with small-scale farmers and local officials in two case study areas.^2^ These interviews sought to understand why and how jatropha had come to be promoted as the primary biofuel crop in Kenya, and how the experiences of farmers growing jatropha compared to the claims of those who had encouraged them to grow it (Hunsberger 2010, 2014). Nguruman and Mpeketoni were chosen as case study sites because both were experiencing a high level of jatropha activity involving small-scale farmers but were different enough to capture contrasting experiences. Mpeketoni involved a centrally organized, European-funded project in which farmers with land titles grew jatropha as a local energy source on part of their land. Nguruman had no formal jatropha project but became a hub of buying and selling seeds collected from existing fences, leading farmers in a group ranch land tenure system to wonder how lucrative further jatropha cultivation might become. Following the 2009 fieldwork I remained in limited email contact with key informants, for example asking them to approve the use of quotations from their interviews, sharing documents I had written, and in a few cases asking for updates about their activities.

In November–December 2013 I returned to Kenya with the aim of re-interviewing as many of the same people as possible. Prior to this visit I contacted most of the original informants by email, using internet searches to investigate whether those who did not respond had changed their affiliation. On arriving in Kenya I followed up with further emails and phone calls. I succeeded in re-interviewing 9 of 23 original key informants (eight in person, one by phone), had informal conversations with another three (one in person, two by phone), and received brief email updates from two others who were out of the country at the time. While key informants’ perspectives are the focus of this paper, I also revisited one of the two case study sites (Nguruman), speaking with about one-third of the farmers and local officials who had participated in the 2009 study (10/27).

The individuals who were re-interviewed were spread across the categories of government (2 individuals), NGOs (2), private sector (2), researchers (2), and donors (1).^3^ The 2013 transcripts and interview notes were coded for the following themes: optimistic and critical statements about jatropha; why jatropha activities had diminished; goals that were initially attached to jatropha; and interviewees’ current activities and goals. The 2009 interview transcripts were re-examined, particularly in relation to actors’ stated motivations for working with jatropha, expected benefits, and perceptions of what factors were influencing jatropha activities in the country. After providing some context about jatropha in Kenya, the following sections explore the content of these interviews.

### Jatropha in Kenya

This section provides a brief history of jatropha biofuel activities in Kenya and considers how this experience compares with other countries where jatropha biofuel projects have occurred.

Like many countries in the global South, Kenya experienced a wave of interest in jatropha as a fuel source in the first decade of the twenty-first century—but the plant has a much longer history in the region. The jatropha plant is indigenous to Latin America and was probably introduced to Africa by Portuguese traders before 1810 (Heller 1996). The oil from jatropha seeds has historically been used for soap making and street lighting, while other parts of the plant have been used medicinally as well as to make inks, dyes and pesticides (Heller 1996; Orwa et al. 2009). Recently, interest in jatropha as an energy source has taken hold in parts of Africa, Asia and Latin America. While some common patterns have emerged, including lower than anticipated long-term job creation, disappointing incomes for small-scale growers and widespread project failure (Romijn et al. 2014), the Kenyan experience may be unique in two respects. First, unlike in India, where a national biodiesel policy encouraged private sector investment and widespread jatropha cultivation (Baka 2014; Ariza-Montobbio et al. 2010), in Kenya NGOs were the primary drivers of early jatropha activities—even helping to draft a biofuel policy document. Second, unlike in Zambia (German et al. 2011a), Madagascar (Neimark 2016), Ghana (Schoneveld et al. 2011), Tanzania (Romijn et al. 2014) and Mozambique (Nicotra 2015), Kenya did not experience major controversies over large-scale plantations until a wide range of projects aimed at small-scale farmers were already underway. This affected the discourses at play in the early stages of jatropha development, with more emphasis on ‘pro-poor’ goals relative to ambitions over national economic development, import substitution and trade.

In 2008–2009 when I began this research project, jatropha was at the centre of a flurry of activity by NGOs, government officials, private companies, researchers and international donors (Hunsberger 2010). Several Kenyan NGOs backed by international funding were promoting jatropha to small-scale farmers as an energy crop. The government had endorsed jatropha as ‘the choice crop’ for biodiesel (Government of Kenya 2008), and had included NGOs, private companies and researchers in a National Biofuels Committee tasked with guiding the development of a biofuel policy, among other duties. Private companies were running pilot plantations and social enterprise projects, though no large-scale plantations had been established. Various research activities were underway, led by government agencies, private consultants, international organizations and universities. By 2009 a research sub-committee of the National Biofuels Committee had formed to coordinate communication between these research efforts. In addition to the National Biofuels Committee convened by the Ministry of Energy (and chaired by the Petroleum Institute of East Africa), a separate entity called the Kenya Biodiesel Association (KBDA) had formed to help promote and facilitate biodiesel production in the country. The membership of these two bodies overlapped considerably, with some of the members also acting as local promoters in direct contact with farmers. For a more detailed introduction to these actors and how they related to each other, see (Hunsberger 2010).

Estimates of how many farmers were growing jatropha in 2009 ranged from under 500 (GTZ 2009) to over 4000, the higher number coming from self-reporting by NGOs (Hunsberger 2014). Whatever the true number, uptake by small-scale farmers reflected a novel wave of interest in jatropha as a source of biofuel at the time. The plant was not new to Kenya: farmers in some areas had already been growing the plant for decades, either as individual trees or as fencing grown from closely spaced cuttings. Some farmers were thus familiar with the jatropha plant before biofuel projects were introduced. However, there was no precedent in the study areas for trying to maximize seed production, extracting oil or selling any jatropha products.

Nguruman, Kajiado County, was one such area that came to be seen as a source of jatropha seeds. Numerous actors purchased seeds from farmers to establish research projects or seedling nurseries, either by visiting the area themselves or by making arrangements through local intermediaries. These buyers offered wildly fluctuating prices that reflected the availability of research and donor money as well as the profit potential of nurseries, rather than prices that could be sustained over the long term if jatropha seeds were used to produce cost-competitive alternatives to diesel or kerosene. Farmers in turn developed widely varying expectations for the benefits that jatropha might bring them. Their hopes ranged from having the opportunity to gain occasional extra income to establishing a processing factory in the area that would employ local youth.

Experiences in Mpeketoni, Lamu County, were markedly different. Here a Norwegian NGO had funded a project in which more than 1000 farmers agreed to grow jatropha on part of their land with the aim of producing energy for local use. Local project coordinators planned to use fuel from jatropha to power grain mills, a cotton ginnery, irrigation pumps, and boats, as well as household lamps and cookstoves. In 2009 farmers were beginning to harvest their first jatropha seeds (despite damage from pests and diseases) and the project faced decisions about how to develop and implement its business plan.

Other notable jatropha activities in 2009 included efforts by the NGO Green Africa Foundation (GAF) to encourage both small-scale farming and investments in large plantations. In 2009 GAF claimed to be working with ‘thousands’ of farmers while also running a seedling nursery and jatropha test farm in Yatta (NGO interview, 2009). The Vanilla Development Foundation and Vanilla Jatropha Development Foundation (which started as a single organization and later split into two) also sold seeds to small-scale farmers in several parts of the country. Two private sector projects were also operating in 2009: an outgrower scheme involving 70 farmers in Shimba Hills, and a pilot plantation in Mwingi run by Better Globe Forestry with an eye toward developing a nucleus-outgrower arrangement in the future.

Between 2009 and 2013 a number of events signalled a decline in enthusiasm and activities related to jatropha in Kenya. Two foreign companies negotiated access to land to establish large plantations, in the Tana Delta and Dakatcha Woodlands, but both projects became embroiled in controversy and the investors’ plans were never realized (Krijtenburg and Evers 2014; Smalley and Corbera 2012; Ross 2011). The biodiesel policy drafted with the input of a multistakeholder committee was not implemented, as the government eventually decided to revise its overall energy policy instead of adopting new policies on specific topics such as biofuels (GOV1 interview, 2013).^4^ Research projects that were underway in 2009 concluded that jatropha yields were “dismal” (Iiyama et al. 2013) and that cultivating jatropha should not be pursued under current conditions (GTZ 2009; Iiyama et al. 2013; Pipal 2012). Pilot projects petered out, including Better Globe’s (PS interview, 2013), and the Kenya Biodiesel Association became largely inactive (NGO3 interview, 2013). Farmers in Nguruman reported that visits from buyers had tapered off. And crucially, the number of NGOs promoting jatropha to small-scale farmers declined: by 2013 the leaders of two of the organizations that had been most vocal in promoting jatropha in 2009 (Vanilla Development Foundation and Vanilla Jatropha Development Foundation) had taken on new jobs outside of these organizations, while a third leading jatropha NGO (Green Africa Foundation) had shifted its focus to other projects. Actors’ explanations of their changes in priorities and activities will be discussed below.

At least two jatropha projects had continued, albeit with a broader focus that included activities other than jatropha. In Shimba Hills, some of the farmers who had participated in an outgrower scheme run by a private company (Energy Africa) continued to grow jatropha in 2013 alongside vegetables and herbs. The project pressed and filtered its own jatropha oil, for which it had found a niche market: a nearby hotel bought the oil to use in floating-wick lamps in its restaurant. In Mpeketoni, the project initiated by Norwegian Church Aid continued to pursue jatropha as a useful crop despite setbacks from low yields, technical obstacles and organizational challenges. The project had given up on one of its initial ideas—to use jatropha oil for household lamps, whose design proved too problematic—and had broadened its focus to include other sources of biodiesel.

### Representations of jatropha

This section explores the representations of jatropha expressed by key actors in Kenya during and after the events described above in order to approach the research questions.

#### Before: 2009

In 2009, jatropha held primary status among biofuel crops in Kenya—a view expressed in policy documents and biofuel-related events, and voiced by many key actors in their interviews. A draft government strategy document produced in 2008 stated that jatropha was the top priority for biodiesel (Government of Kenya 2008), a position borne out through interviews. One government representative described jatropha as ‘a better candidate than any other’ for biofuel production in Kenya because of its potential to produce energy in ‘marginal’ areas without compromising food security (GOV1 interview, 2009). Jatropha’s dominant status was also apparent at a June 2009 research forum called the Kenya Biodiesel Workshop, whose content focused almost entirely on jatropha (Ministry of Energy 2009).

NGO representatives reinforced jatropha’s relative importance in 2009. One stated: ‘The [government’s] biofuel strategy is basically the jatropha strategy. There’s no other oil that’s seen to come close’ (NGO4 interview, 2009). Notably, this individual’s organization was a member of the National Biodiesel Committee that gave input to the government’s 2008 draft biofuel strategy, and was identified by other interviewees as one of the most vocal advocates of jatropha in Kenya. Another NGO representative (who had a more ambivalent position on jatropha and was not part of the National Biodiesel Committee) commented, ‘Jatropha is a bandwagon. We’re on it; even you are on it now. We don’t know where the wagon is going but the band is playing’ (NGO8 interview, 2009). These statements illustrate a strong discourse of jatropha being the most important—perhaps even the only important—source of biofuel in the country at the time.

However, in 2009 key actors already acknowledged challenges and several spoke about the need to consider alternatives to jatropha. In some cases disappointing experiences had reduced their initial enthusiasm. One representative of a donor organization said, ‘As soon as we started planting [jatropha] and getting a bit more research on it, and getting more feedback from the farmers, it became increasingly clear that it may not be that wonder crop that people present it to be. For sure we have our own reservations about whether jatropha can work as a plantation crop’ (DN1 interview, 2009). These observations did not deter the organization from pursuing the project they had started at the time. Similarly, a private sector actor stated that although the jatropha project they were involved with had achieved something in terms of producing oil, learning, building relationships, and gaining recognition through partnerships, ‘In terms of what our expectation was, the achievements are minimal and the progress is extremely slow and disappointing’ (PS2 interview, 2009). These comments show early recognition that efforts to produce energy from jatropha were overly optimistic. Regarding alternatives, a researcher who was involved in preparing the government’s draft renewable energy strategy of 2008 reflected, ‘initially the strategy was focusing on jatropha alone to promote the industry. But later on we realised that there were quite a number of other feedstocks which could actually be very useful and there’s need for diversification’ (RS4 interview, 2009). This comment echoed others made in 2009 that jatropha alone was unlikely to reach energy production goals.

Research results were also beginning to temper expectations in 2009. One study presented at the research workshop mentioned above (later published as GTZ 2009; Iiyama et al. 2013) suggested that growing jatropha as fencing was the only strategy that could provide modest economic benefits over the plant’s lifespan, while growing it in fields, either as a monoculture or intercropped, would produce negative economic returns. While some actors resisted the dissemination of these findings, in 2009 evidence was beginning to accumulate suggesting that jatropha’s prospects were lower than many had hoped. This outlook resonated with some informants’ lack of confidence in the evidence available from other countries were jatropha cultivation was underway. For example, one private sector actor lamented: ‘I rarely find a real, scientific article on the internet which gives all the information you need to apply it here’ (PS3 interview, 2009).

Despite these early signals, individuals working with the crop in 2009 identified a wide range of goals they hoped it would achieve—and took numerous actions to promote and protect a positive image of the crop. The goals they associated with jatropha included increasing forest cover while generating livelihood benefits, providing a source of income in dryland areas, and producing clean energy for the country while alleviating poverty (see Table 1). While it was common for these informants to link together at least two goals, some went further: in Mpeketoni, project coordinators hoped jatropha would reduce farmers’ household expenses by allowing them to grow their own energy, improve educational opportunities by fueling household lamps, raise incomes by providing energy for irrigation, crop processing and value addition, and provide transportation fuel for boats. ‘Bundling’ multiple objectives together in this way increased the breadth and depth of jatropha’s appeal, a theme to which I will return.

**Table 1:** Comparison of stated goals and activities for select organizations working with jatropha in Kenya, 2009 and 2013

Organization	2009 Stated goals (activities)	2013 Stated goals (activities)
Better Globe Forestry	Dryland trees for income (jatropha pilot, plans to expand)	Dryland trees for income (other trees—no jatropha)
Practical Action	Rural household energy (researching jatropha among other options)	Rural household energy (ethanol cookstoves—no jatropha)
Energy Africa	Farmer income; household energy source (70 jatropha outgrowers)	Farmer income (selling jatropha oil to hotel; also vegetables and herbs)
Green Africa Foundation	Forest cover; livelihood benefits (promoting jatropha to small farmers and large investors)	Rural livelihoods; climate change (sanitation projects; solar lanterns)
Ministry of Energy	Poverty alleviation; rural energy access; national fuel blending (endorsed jatropha as priority biofuel crop)	Moving rural people up the energy ladder to clean energy (still considering jatropha for local use, not large-scale)
Mpeketoni 1	Fuel for lamps, pumps, generators, crop processing, transportation; carbon credits (1000+ farmers growing jatropha)	Energy for local use; skin product (supplementing jatropha with castor; selling jatropha oil in small quantities)
Mpeketoni 2	Fuel for lamps, pumps, generators, crop processing, transportation; carbon credits (1000+ farmers growing jatropha)	Energy for local use; edible oils; consumer products; livestock feed (promoting cotton—not jatropha)

One way that actors sought to directly shape discourses about jatropha was to publicly air their perspectives through the media. At least two key actors wrote opinion pieces in newspapers that were published under their own names, while two others were quoted extensively in newspaper articles written by reporters. One NGO leader expressed an ambition to launch a TV and radio station to spread ‘proper information’—meaning, to have greater control over the messages being circulated. Notably, actors used media coverage both to promote and to critique the idea that jatropha was a ‘wonder crop.’

Some key actors handled dissent by speaking harshly about other individuals or organizations, acting to discredit those with whom they disagreed. One researcher criticized the motives of NGOs who were funded to work on jatropha and sold seeds to farmers, saying, ‘they’ve gamed the system. They’ve taken advantage of people’ (RS1 interview, 2009), referring to NGOs that sold seeds to farmers and did not return to help them cultivate jatropha successfully, buy back their harvest or help develop local processing capacity. An NGO actor concurred: ‘People are promoting jatropha only because they have donor money to do it, with the result that farmers suffer’ (NGO1 interview, 2009)—again referring to farmers left without support or markets for a new product. Another NGO actor sharply criticised other NGOs for making false promises, calling them ‘quacks’ who ‘cheat’ farmers by telling them they would be able to sell jatropha seeds for a high price (NGO2 interview, 2009). NGOs were not the only ones to come under attack: key informants also alleged that government processes lacked fair representation, researchers were guarding information, and private investors were engaging in bribery. What was striking about these grievances was the personal tone with which they were delivered: not only did actors criticize others’ activities or decisions; they also made harsh inferences about their motivations and character.

A second strategy for maintaining positive representations of jatropha was to bundle together a set of expected benefits that were likely incompatible. Project leaders in Mpeketoni talked about a large number of products that could be made from jatropha (e.g. lamp oil, cooking fuel, electricity, biodiesel for transportation, soap, and carbon credits) when realistically, a project would likely have had to choose between these outputs rather than produce them all simultaneously. As well, several NGO actors spoke about large-scale and small-scale jatropha production being mutually dependent, implying that the pursuit of jatropha in Kenya would automatically achieve both the national economic goals associated with large-scale monoculture production and the poverty alleviation and community development goals associated with small-scale production. Conflating multiple goals in this way gave the impression that jatropha projects were more robust, resilient, and capable of producing more diverse benefits for a broader range of people than they were likely to achieve—and created opportunities to strategically maneuver between discourses depending on the situation.

The first round of this research therefore found that jatropha’s promoters in Kenya not only used the ‘sustainable biofuel’ discourse to sustain public, political and financial support, they also tailored their representations of jatropha to suit different situations. This ‘discursive flexibility’ helped further insulate jatropha against critique (Citation removed for blind review). For example, initially representing jatropha as a scaffolding plant for vanilla vines and later as a source of energy helped keep farmers committed to growing jatropha after the first objective failed. Conflating goals that could be achieved through large plantations and community-based projects also helped to build and maintain momentum for jatropha activities, likely delaying the impact of emerging research that cast jatropha in a negative light (e.g. GTZ 2009).

#### After: 2013

By 2013, key actors’ representations of jatropha in Kenya were noticeably less optimistic than they were in 2009. All of the informants who were re-interviewed agreed that both the level of enthusiasm and the extent of jatropha activities in the country had dropped off in the intervening years, saying for example, ‘the interest [in jatropha] has gone down very, very much’ (RS4 interview, 2013), ‘it is forgotten for now’ (GOV2 interview, 2013), and ‘we are still pursuing [jatropha] but at a very low level, that’s for sure’ (NGO3 interview, 2013).

Informants gave a variety of explanations for why jatropha activities and optimism had diminished. A commonly cited reason was that the plant simply didn’t grow well: it was attacked by pests and diseases, produced low yields under dry conditions, and even produced low yields under wet conditions (PS3, NGO10, RS4, DN1 interviews, 2013). Where it did grow, it was too labour-intensive to weed, harvest and shell the seeds (NGO10, DN1, PS2 interviews, 2013). Several spoke of a lack of market development, for example saying that the level of investment had remained too low to build momentum for the nascent sector (GOV2, NGO2, NGO3 interviews, 2013). Put another way, one researcher stated that ‘the champions, the people who were really talking about jatropha from the very beginning, didn’t take their story to completion’ by developing markets (RS4 interview, 2013). Two lamented the lack of policy implementation, believing that a favourable biofuel policy could have given farmers and investors more confidence (NGO3, RS4 interviews, 2013). Some stated that not enough research had been done to put jatropha projects on a firm footing; activities had run ahead of the information that was available (GOV4, NGO3, NGO10 interviews, 2013). Finally, one blamed bad publicity stemming from controversies over proposed large-scale plantations in Tana Delta and the Dakatcha Woodlands for driving down public interest (NGO3 interview, 2013), while a former NGO leader blamed ‘negative publicity about NGOs’ more generally (NGO4 interview, 2013). Thus, key actors attributed jatropha’s disappointing results both to ecological factors that they perceived as originating outside the sphere of human influence (agronomy, climate constraints, pests) and to events directly stemming from human decisions (lack of policy, lack of market development, acting without enough knowledge, bad publicity).

Several informants from 2009 had changed their roles by 2013. Two had moved to other parts of the country, also changing their sector of employment (NGO to government; NGO to academia). Two had left the country and changed their thematic focus. Others had remained in the same location and sector but changed their role (moving within government) or their activities (substituting new projects on different themes). Those who had stayed in the same posts with the same mandates (e.g. government positions in energy or forestry), or had remained committed to long-term projects were most likely to have remained involved with jatropha in some way. Those who had moved on to completely different pursuits—geographically, thematically and organizationally—were the hardest to re-interview; it is a limitation of this study that their perspectives are under-represented.

Those who had remained in the same roles and changed their focus from jatropha to other activities gave a range of explanations for why they did so. At least two organizations had maintained a consistent, specific goal in the 2009 and 2013 interviews, finding other means to approach the same goal as they moved away from jatropha. One of these was a private company, Better Globe Forestry, that consistently identified their mandate as promoting income-generating trees in dry areas. After struggling to produce jatropha seeds in a profitable way, the company decided in 2010 that other tree species, such as acacia or gum arabic, were more economically promising for drylands and pursued these instead. Another organization, Practical Action, had initially been interested in jatropha as a source of rural energy for household use. By 2013 they had decided that ethanol cookstoves showed greater potential than biodiesel to improve rural energy access and had shifted their focus accordingly (see Table 1).

As explained above, many who worked with jatropha in 2009 expressed hope that it would achieve a whole set of goals simultaneously. One of the initial coordinators of the Mpeketoni project was no longer involved with it in 2013, but applied the same pattern of thinking in promoting cotton as another multi-purpose crop. But by 2013 several interviewees showed signs of disentangling their ‘bundled’ objectives and choosing one or more to prioritize. For example, one NGO that had actively promoted jatropha in 2009 as a means to expand forest cover while providing livelihood benefits (Green Africa Foundation) had in 2013 dropped its focus on trees. A representative of this NGO explained that they were now pursuing goals related to farmer livelihoods and climate change through sanitation projects and solar lanterns respectively, adding that these activities represented “low fruit” that encountered less resistance than jatropha projects.

The Mpeketoni project had also gone through a process of ‘unbundling’ its initial suite of goals. By 2013 the project had abandoned its idea of burning jatropha oil in household lamps after trying several unsuccessful lamp designs, and had given up on obtaining carbon credits because the administrative requirements were too onerous both in terms of time and money. These decisions signalled a narrowing of ambitions related to jatropha.

Nonetheless, the Mpeketoni project continued trying to produce biodiesel, making up for jatropha’s shortfall by adding another energy crop. A coordinator explained that the project remained committed to helping farmers grow an energy source that could enable them to process and therefore add value to other agricultural crops—but that jatropha alone was not likely to achieve this goal, so they were beginning to grow castor alongside it. This strategy represented an effort to supplement jatropha with other means in order to keep pursuing one of the project’s original goals. One geographic feature in particular may have influenced the persistence of jatropha growing in Mpeketoni: due to the region’s climate and agricultural history, Mpeketoni farmers were already used to cultivating fruit and nut trees before the jatropha project began.

A similar pattern emerged for Energy Africa, a small company in Shimba Hills. In 2009 around 70 farmers were engaged as jatropha outgrowers (down from an estimated 200 at the project’s peak). At that time the coordinator described jatropha as a cash crop with an unlimited market that farmers could use themselves if prices dropped. By 2013, most of Energy Africa’s activities focused on producing and selling vegetables and herbs, but the coordinator reported that jatropha remained worthwhile as one activity among many for farmers who had stuck with it. The project owned a diesel-operated press and sold jatropha oil to a nearby hotel for use in floating-wick lamps in its restaurant. This niche market allowed the group to generate modest income. In addition to selling jatropha oil, Energy Africa was using jatropha press cake as fertilizer mixed with grass, soil and manure, and some farmers had found that passion fruit plants climb well on jatropha. Jatropha had shrunk in importance relative to other farming activities, but remained viable as a sideline activity thanks to the presence of a unique local buyer. This project’s spatial location played a key role in establishing a market for jatropha oil: its proximity to a coastal area with a thriving tourism sector allowed it to link with a hotel that could afford to buy jatropha oil at favourable prices, an opportunity not available in other areas of the country.

Farmers in Nguruman, who in 2009 hoped that jatropha could generate income and perhaps even support a local processing factory, reported in 2013 that local people had very little interest in collecting jatropha seeds anymore. Some said there had been no buyers for a long time; others said that they had heard about one or two offers to buy seeds within the past 6 months but the prices offered were too low. Interviewees in Nguruman most frequently blamed the lack of a market and a lack of leadership for jatropha’s failure to deliver on expectations. While jatropha plants proved able to survive in Nguruman’s climate with relatively little care, other aspects of the area’s spatial and social geography may have worked against the success of jatropha activities: the road from the nearest town to the community is in poor repair, passes through very rugged terrain and has only one slow and crowded bus making a daily journey, thereby discouraging the easy movement of people and goods. A historic lack of coordination between farmers and a low level of trust in local agricultural officials likely compounded this situation.

A government representative acknowledged in 2013 that Kenya did not yet have a working jatropha project and cautioned against pursuing large-scale activities, but continued to consider jatropha’s potential to supply biodiesel for rural development and electricity generation. This individual emphasized that ‘unclean energy’ was still a major issue in Kenya; the country’s energy problem had not gone away even though jatropha had been disappointing. This view represented consistent commitment to one goal—expressed in 2013 as ‘helping rural communities rise up the energy ladder to clean energy’—even as it signalled a retreat from an earlier hope that jatropha might support a national fuel blending mandate.

To summarize, while one former project coordinator responded to jatropha’s poor results by looking for a new crop that could satisfy a similar bundle of objectives, most informants responded by scaling back their ambitions and ‘unbundling’ their initial hopes for jatropha back into separate parts. Some sought alternative ways to pursue these narrower goals separately, dropping jatropha altogether, while others tried to supplement jatropha with additional activities to keep pursuing a subset of their initial goals.

## Discussion

This paper’s first objective was to explore how actors explained jatropha’s story after disappointing results. In 2009, despite some cautionary statements, informants predominantly expressed high hopes and multiple, often conflated expectations for jatropha. This optimism reached such an extent that several informants described and defended jatropha as though it were an end in itself rather than a means to reach other goals (Hunsberger 2010). By 2013, a few actors maintained that jatropha could have met expectations if different conditions had been created—if a policy had been put in place, or if critics had not received so much attention—but most had substantially changed their views about what it was possible to achieve with jatropha. The 2013 interviews revealed general agreement that the level of interest and activities dedicated to jatropha in Kenya had gone down, and key actors expressed lower expectations for jatropha’s potential in 2013 compared to 2009. Interviewees attributed this decline to both what they interpreted as extra-human biophysical factors (poor growth due to climate, pests) and to human decisions (lack of policy, knowledge and markets; bad publicity).

The second objective was to investigate whether and how actors changed their representations of jatropha over time. Patterns emerged in the ways that actors explained their goals in 2013 and how they linked these with the expectations and strategies for jatropha they expressed in 2009 (Table 1). Initial visions of using jatropha to combat climate change, alleviate rural poverty or provide clean energy for development were either transferred (e.g. to cotton, another multi-purpose crop) or transformed into other projects (e.g. solar lanterns). Both strategies show traces of the same discourses initially used to promote jatropha—either intact or fragmented into smaller pieces—being re-used to advance other projects after jatropha’s demise. While one informant continued to conflate multiple goals to the same extent in 2013 as in 2009, most opted instead to choose one or two of the goals jatropha was originally supposed to achieve and looked for another way to approach it by pursuing alternatives or supplementary means. These developments suggest that in the eyes of many of its initial promoters, by 2013 jatropha had moved back to being one of several possible means to reach various ends rather than an end in itself.

This analysis offers insights back to theory in two areas: on the economic power of discursive representations and on responsible innovation. Regarding the first, this paper began by considering ideas about the ability of discourse to shape economic conditions and lived experiences. Tsing (2000), Jessop (2005), and Fairhead et al. (2012) all speak to the general potential for speculation about future gains to help create present economic conditions that favour a particular undertaking. For jatropha specifically, Neimark (2016) highlights how development discourses helped bring contentious (and short-lived) jatropha projects into being in Madagascar, while Vel (2014) writes that knowledge ‘brokers’ played a key role in boosting jatropha’s status as a ‘discursive commodity’ in Indonesia, turning expectations about potential future profits into present objects of trade by selectively relaying incomplete information. The case examined here suggests that positive representations of jatropha sustained a period of optimism about the crop longer than experience alone could have done, giving advocates time to enroll more farmers in growing jatropha. Ultimately, though, the favourable representations that dominated in 2009 did not succeed in producing the economic conditions they predicted. By 2013 many in Kenya had lost faith in jatropha’s potential to realize its anticipated value, suggesting that jatropha oil did not successfully make the transition from a discursive commodity into a physical product with a functioning market This resonates with Afiff’s (2014) observation that jatropha experienced a ‘hype-disappointment’ cycle (based on expectations alone) rather than a ‘boom-bust’ cycle (where a market develops and then fluctuates or crashes). This is not to say that jatropha’s presence in Kenya was ‘only’ discursive. Quite the contrary, jatropha as a plant, a living fence and a source of traditional medicine was already woven into the lives of many rural people before interest in biofuels arose. It is jatropha as a fuel that has only materialized in limited, specific circumstances (such as in Shimba Hills).

If positive representations of jatropha did extend the length of time that an unviable project was seen as viable, this raises questions about responsible innovation. For example, at what point should it have become clear that jatropha was unlikely to live up to expectations? Tsing’s (2000) initial analysis of the ‘economy of appearances’ focused on Bre-X, a mining company that attracted investment through fraudulent claims of a major gold find—a case that highlights the potential for positive representations to prop up ultimately harmful enterprises. Unlike Bre-X, jatropha’s promoters did not act on willful deception from the start. The outcomes of innovation are by definition unclear at the outset, and all new ideas rely on positive early publicity—including those that are later regarded as successful (for example, mobile phone banking in Kenya). By prolonging harmful experiments or supporting beneficial ones, lofty promises can thus either improve or worsen outcomes depending on the innovation.

How, then, might unsuccessful strategies be detected earlier so that the worst of their impacts can be avoided? To address this question we can look for early indicators that jatropha would turn out to be an innovation failure instead of a success—indicators that might help inform decisions about other innovations. With hindsight, the lack of solid research about jatropha, the fact that most organizations skipped pilot testing in their enthusiasm to try something new, and the relatively strong presence of doubtful counter-messages even at the time of peak interest in jatropha appear as warning signs.

The case of jatropha in Kenya also invites reflection on who should take responsibility for the negative impacts of an unsuccessful innovation—and what it means to do so. Tempels and Belt (2016) describe how difficult it is to assign responsibility for the indirect consequences of biofuel production, cataloguing for example how US biofuel industry groups tried to sidestep responsibility for indirect land use change by questioning the techniques used to measure it. Research in Kenya and elsewhere suggests that NGOs, local officials, knowledge brokers, researchers and entrepreneurs have benefited most from experiments with jatropha, while farmers and farm workers have carried the bulk of the risks and most often ended up with little reward (German et al. 2011a; Neimark 2016; Vel 2014; GTZ 2009). Without any mechanisms specifying responsibilities that jatropha innovators had towards those who were likely to be affected by their projects, decisions about whether and when to walk away from jatropha projects could be made based on a sense of accountability to donors (for NGOs), shareholders (for private companies), funders (for researchers) and the electorate (for government)—as well as the individual goals and aspirations of those involved. Thinking systematically about how such actors interpret and act on a sense of responsibility could provide a starting point for approaching more difficult questions of who should do what to mitigate the impacts of an innovation gone wrong.

Key actors who were interviewed in this study differed in their decisions about whether to continue pursuing or to abandon a disappointing experiment. There were NGOs and private companies that followed each path. If one views these choices as expressions of organizational self-interest, one could conclude that some prioritized the agility that comes with pursuing new opportunities while others chose the reputational stability that comes with sustaining a long-term commitment. Read another way, one could interpret the same choices as expressions of where these organizations located their sense of responsibility: some seeing it as more responsible to adapt to changing knowledge by exchanging jatropha for more promising activities; others seeing it as more responsible to continue seeking ways for farmers who had already incurred the costs of cultivating jatropha to benefit from it in some way. Government officials occupied an intermediate position by remaining open to the possibility that jatropha might offer a partial solution to broader problems they were mandated to solve, such as expanding energy access and mitigating deforestation. With little flexibility to redefine their goals, government actors’ sense of responsibility appeared to be located within a commitment to these wider issues, with jatropha remaining on the table due to a lack of obviously preferable alternatives. Further work examining key actors’ perceptions of their individual and organizational responsibilities in more detail could provide ideas about how to leverage this sense of responsibility to protect those most vulnerable to negative impacts from untested innovations, and how to redress harm where it has occurred.

## Conclusions

This paper has explored an example of how biofuel projects are constructed, negotiated and renegotiated over time. Drawing on two sets of interviews, it documents how individuals who were involved with early jatropha activities in Kenya explained the crop’s story and framed their activities before and after disappointing results. By comparing these informants’ statements in 2009 and 2013, we see how representations that were initially used to propagate jatropha continued to be put to work—sometimes in the service of similar biofuel activities, sometimes to promote very different ventures.

Several interviewees provided evidence that ‘bundled’ representations of jatropha—statements that rolled together energy, climate change and rural development goals—had been recycled to advance projects with many similarities. For example, efforts to produce biodiesel or household fuel from other agricultural sources drew on the same set of ambitions as the jatropha projects that came before them. In Mpeketoni, a former project coordinator who was disappointed with jatropha’s results had begun to pursue another multi-purpose crop: cotton. These cases represent actors holding onto similar combinations of goals while trying different means to achieve them.

In other cases actors ‘unbundled’ their initial visions of jatropha, directing their efforts toward new projects that shared one or more features of the jatropha agenda while letting others fall away. Examples include promoting solar lanterns for household use, a strategy that seeks to increase access to clean energy but drops initial goals related to farmer incomes and reforestation. Similarly, sanitation projects aim to improve farmer livelihoods in ways unrelated to energy, climate change or agriculture, while selling jatropha oil to a local hotel supports the goal of generating income for farmers but does not produce energy for household use. These strategies reflect actors disentangling multiple objectives and making efforts to pursue them separately.

‘Unbundling’ the objectives associated with an innovation like jatropha makes pragmatic sense after the initial suite of goals has proved difficult to achieve. But it would also be a useful proactive exercise—to explicitly look for tensions and trade-offs between ‘bundled’ goals and assess how realistic it is to think that a new innovation might reach any of them, let alone all. Some have approched this task for jatropha in other ways, for example finding incompatibilities between the different kinds of ecosystem services jatropha has been claimed to be able to provide and the scales of cultivation that could realistically produce them (van der Horst et al. 2014).

One may ask whether the response strategies described here for a failed biofuel crop might also apply to an even broader set of ambitions: emerging expectations for the bioeconomy. While biofuel crops like jatropha have been linked to a diverse suite of goals, discourses of the bio-economy expand the range of possibilities even further to include producing chemicals, pharmaceuticals, plastics and other materials from plant sources (McCormick and Kautto 2013). It seems plausible that hoping for multiple, simultaneous value chains to develop and multiple benefits to flow from single feedstocks—particularly if, like jatropha, these feedstocks are under-tested or expected to grow in ‘marginal’ conditions—could lead to either an acceptance of trade-offs (realization that some goals can be achieved but not others) or a constant quest for the next thing that might reach all goals at once.

 What are the consequences of these two strategies? Substituting new multi-purpose crops when an old one falls short of expectations could become an endlessly repeating cycle. Decisions about how to break down a multi-purpose crop into separate ways to achieve separate things may end up conforming to economic pressures to pursue the products that generate the most financial reward—at the expense of some of the ideals of the bioeconomy. The future of cotton in Mpeketoni and of other post-jatropha projects elsewhere may provide clues of what is to come if the search for new ‘silver bullets’ and the strategic narrowing of objectives accompany efforts to pursue a plant-based economy.

## Footnotes

1 In this work I interpret discourse broadly as a paradigm or narrative that includes language as well as the assumptions and representations through which language is translated into social meaning (Grillo 1997). For the sake of simplicity I use the terms discourse and representation here, recognizing that political ecologists have also used other terms such as narratives (Fairhead and Leach 1997), imaginaries (Neimark 2016), perceptions and competing knowledge claims (Bryant 1998; Walker 2005; Mung'ong'o 2009) to refer to the ways in which understandings of environmental change, development and access to resources are socially constructed and enacted.

2 The 2009 research was approved by Carleton University’s Research Ethics Committee. Consent was obtained from all research participants in both 2009 and 2013. I have done my best to protect the identities of the individuals involved.

3 Some informants had overlapping roles that spanned more than one category. In these cases I assigned them to what I considered to be their primary category.

4 To avoid identifying interviewees I refer to them using codes. Their affiliations are abbreviated as DN (Donor), GOV (Government), NGO (Non-governmental organization), PS (Private sector), and RS (Researcher).
